# miR-33a-5p in small extracellular vesicles as non-invasive biomarker for oxaliplatin sensitivity in human colorectal cancer cells

**DOI:** 10.1016/j.bbrep.2021.100996

**Published:** 2021-04-07

**Authors:** Shota Tanaka, Mika Hosokawa, Takumi Miyamoto, Aiko Nakagawa, Mika Haruna, Kumiko Ueda, Seigo Iwakawa, Ken-ichi Ogawara

**Keywords:** Extracellular microRNA, Small extracellular vesicles, Oxaliplatin, Biomarker, Liquid biopsy, Colorectal cancer

## Abstract

microRNAs (miRNAs) contained in small extracellular vesicles (sEVs) are candidates for non-invasive biomarkers. Oxaliplatin (L-OHP) has been approved for advanced colorectal cancer (CRC) chemotherapy. However, the response to L-OHP differs among CRC patients. In addition, CRC cells often acquire the resistance to L-OHP. This study aimed at the prediction of L-OHP sensitivity by measuring extracellular miRNAs levels. Firstly, we compared intracellular miRNAs expressions in L-OHP-sensitive CRC cells (SW620 and HCT116 cells) with those in acquired and intrinsic L-OHP-resistant cells. In microarray and real-time RT-PCR analyses, the intracellular miR-33a-5p, miR-210–3p, and miR-224–5p expressions were lower in acquired and intrinsic L-OHP-resistant CRC cells than sensitive cells. Furthermore, in SW620 cells, L-OHP sensitivity was decreased by miR-33a-5p inhibitor. On the other hand, miR-210–3p or miR-224–5p inhibitor did not affect L-OHP sensitivity in SW620 cells. Secondly, the amount of miR-33a-5p, miR-210–3p, and miR-224–5p in sEVs was compared. The amount of miR-33a-5p and miR-210–3p in sEVs secreted from acquired and intrinsic L-OHP-resistant cells tended to be small. miR-224–5p was not detected in sEVs secreted from three types of CRC cells examined. To the best of our knowledge, this is the first study demonstrating that miR-33a-5p and/or miR-210–3p in sEVs would be candidates for biomarkers of L-OHP sensitivity. In particular, miR-33a-5p is a promising candidate because it would be directly involved in L-OHP sensitivity.

## Highlights

• miR-33a-5p was down-regulated in oxaliplatin-resistant colorectal cancer cells.
• Reduction of intracellular miR-33a-5p expression caused oxaliplatin resistance.
• miR-33a-5p amount in small extracellular vesicles from resistant cells was small.
• miR-33a-5p is a candidate for non-invasive biomarker of oxaliplatin sensitivity.

## Graphical abstract

**Figure:**
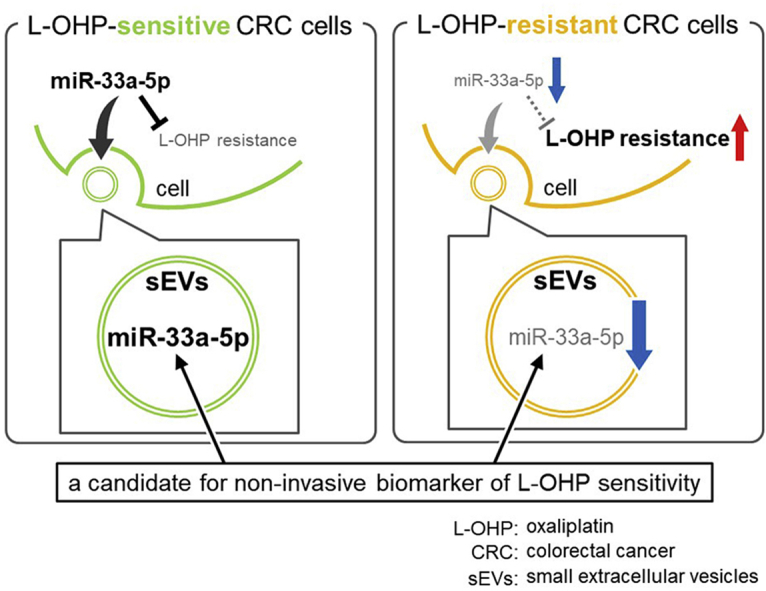

## 1 Introduction

MicroRNAs (miRNAs), a class of small non-coding RNA, suppress mRNA and protein expression. Intracellular miRNAs can cause mRNA destabilization and/or repression of translation through binding to partially complementary sequence in the 3′‐untranslated regions of target mRNA. The several miRNAs play important roles in many biological processes [[1], [2], [3]]. In addition, recent studies have reported that abnormal expressions of intracellular miRNAs were often observed in cancer cells resistant to anti-cancer drugs [[4], [5], [6], [7], [8], [9]].

Colorectal cancer (CRC) is the second leading cause of cancer-related deaths in worldwide [10]. Chemotherapy is the most viable option for the patients with CRC. Oxaliplatin (L-OHP), a third-generation platinum-based anti-cancer drug, has been approved for the first-line chemotherapy of advanced CRC, such as FOLFOX, a combination therapy with L-OHP, 5-fluorouracil, and leucovorin. However, the response to L-OHP differs among CRC patients. In addition, long-term treatment with L-OHP often causes the acquisition of resistance to L-OHP by various mechanisms, including the abnormal miRNA expressions [6,8,9,[11], [12], [13], [14]]. Therefore, the identification of valid biomarkers for the prediction of L-OHP response would be of great importance. Additionally, biopsy of CRC specimens for long-term monitoring of resistance development is needed during chemotherapy but is a heavy burden for patients. Thus, non-invasive biomarkers for acquisition of resistance to L-OHP are urgently required.

Liquid biopsy, the analysis of biomarker in body fluids such as blood, urine, or saliva, is a non-invasive and easy method to monitor disease status of patients at any time point. miRNAs contained in small extracellular vesicles (sEVs) with diameter of 30–100 nm, such as exosomes, are useful samples for liquid biopsy. sEVs are secreted from various cells into extracellular space and contain proteins, lipids, mRNAs, and miRNAs depending on the secreting cells. sEVs are highly stable in body fluids and are also actively secreted from cancer cells [15,16]. Thus, sEVs and these contents have potential as attractive non-invasive biomarkers for cancer diagnosis and the prediction of therapeutic effects. For instance, Ogata-Kawata et al. reported that seven miRNAs in serum may be useful non-invasive biomarkers for CRC diagnosis [17]. In addition, miR-34a-5p in serum sEVs was reported to be a candidate of novel biomarker to predict docetaxel sensitivity [18]. However, extracellular miRNA that is substantially determining the sensitivity of cancer cells to L-OHP has not been identified yet.

This study was aimed to search for the non-invasive biomarkers for predicting L-OHP sensitivity, and two L-OHP-resistant CRC cells were established for this purpose. Moreover, the amount of several miRNAs in sEVs secreted from acquired and intrinsic L-OHP-resistant CRC cells was examined.

## 2 Materials and methods

### 2.1 Cell culture

Human CRC cell line SW620 was purchased from the American Tissue Culture Collection (Manassas, VA, USA). Human CRC cell lines HCT116 and SW480 were purchased from DS Pharmabiomedical (Osaka, Japan). L-OHP-resistant SW620 (SW620-OxR) cells were prepared as previously reported [9]. SW620, SW620-OxR, and SW480 cells were grown in Leibovitz's L-15 medium (Life Technologies Corp., Carlsbad, CA, USA) plus 10% fetal bovine serum (FBS) (Sigma-Aldrich, St. Louis, MO, USA), 100 U/mL penicillin, and 100 μg/mL streptomycin at 37 °C in 100% air. SW620-OxR cells were grown with 80 μM L-OHP (Tokyo Chemical Industry Co., Tokyo, Japan) and were cultured without L-OHP for 1 passage before experiments. HCT116 cells were grown in McCoy's 5A medium (Life Technologies Corp.) plus 10% FBS, 100 U/mL penicillin, and 100 μg/mL streptomycin at 37 °C in 5% CO_2_ air.

### 2.2 Establishment of L-OHP-resistant HCT116 cells

To establish L-OHP-resistant CRC cells, HCT116 cells were cultured with L-OHP for approximately 5 months. HCT116 cells were seeded in 100-mm dish at 3.0 × 10^3^ cells/dish and cultured with 0.8 μM L-OHP. After 14 days, the surviving cells were subcultured with 1.6 μM L-OHP in 100-mm dish [9]. The concentration of L-OHP was increased in a stepwise manner to 1.6 μM (~day 34), 3.2 μM (~day 77), 6.4 μM (~day 108), and finally 12.8 μM (~day 144).

### 2.3 Cytotoxicity assay

The cytotoxicity of L-OHP in cultured cells was evaluated by the WST-8 assay using Cell Counting Kit-8 (Dojindo, Kumamoto, Japan). The cancer cells were seeded in 96-well plate at 3.0 × 10^3^ (SW620, SW620-OxR, and SW480 cells) or 2.0 × 10^3^ cells (HCT116 and HCT116-OxR cells) and were treated with L-OHP for 72 h. The viabilities were expressed as a percentage of the absorbance measured in each cell cultured in normal medium. The calculation of IC_50_ value was performed using the sigmoid inhibitory effect model by the nonlinear least-squares fitting method (Solver, Microsoft Excel 2016) [19]; E = E_max_ × C^γ^/(C^γ^+IC_50_^γ^), where E: the surviving fraction (% of non-L-OHP treated cells); E_max_: the maximum surviving fraction; C: the L-OHP concentration in medium; γ: the sigmoid factor; and IC_50_: the L-OHP concentration producing 50% of E_max_.

### 2.4 Intracellular miRNA expression

Intracellular RNA was extracted by Sepasol-RNA I Super G (Nacalai Tesque, Kyoto, Japan) according to the manufacturer's protocol. One RNA sample each was submitted to Takara Bio Inc. (Shiga, Japana) for human miRNA microarray analysis using Agilent Expression Array (Agilent, Santa Clara, CA, USA). Real-time RT-PCR was performed by Mir-X miRNA First Strand Synthesis Kit (Takara Bio Inc.). In analysis of microarray and real-time RT-PCR, the expression level of miR-103a-3p in each sample was used as an internal standard for normalization of intracellular miRNA expression levels [20,21]. The primer sequences were listed in Supplementary Table 1.

### 2.5 Transfection of miRNA inhibitor into cells

SW620 cells were reverse-transfected with miRNA inhibitor Negative Control #1, miR-33a-5p, miR-210–3p, or miR-224–5p inhibitor (Bioneer, Seoul, Korea) at a final concentration of 10 nM. The transfections were carried out using antibiotic-free Opti-MEM medium (Life Technologies Corp.) with Lipofectamine RNAiMAX Transfection Reagent (Invitrogen). Following 72-h transfection, intracellular RNA samples were isolated from miRNA inhibitor-transfected cells, or these cells were further incubated with various concentrations of L-OHP for 72 h and the viability of these cells was measured by WST-8 assay.

### 2.6 Isolation of sEVs

CRC cells were seeded in 100-mm dish at 1.2 × 10^6^ cells (SW620 and SW480 cells), 1.8 × 10^6^ cells (SW620-OxR cells), 4.0 × 10^5^ cells (HCT116 cells), or 6.0 × 10^5^ cells (HCT116 cells-OxR), were cultured for 4 days and were further incubated in serum-free Leibovitz's L-15 medium (SW620, SW620-OxR, and SW480 cells) or McCoy's 5A medium (HCT116 and HCT116-OxR cells) for 48 h. Cell culture medium was centrifuged at 500×*g* for 5 min at 4 °C to remove detached cells, and then centrifuged at 16,500×*g* for 20 min at 4 °C to remove cell debris using high-speed micro centrifuge CF16RN (Hitachi, Tokyo, Japan) [17]. The supernatant was passed through a 0.20 μm filter and was concentrated by 100 kDa NMWL Amicon Ultra-15 Centrifugal Filter Unit (Millipore, Billerica, MA, USA) [22]. The concentrate was ultracentrifuged at 120,000×*g* for 90 min at 4 °C by Optima TL 100 Ultracentrifuge (Beckman Coulter, Brea, CA, USA). The resultant pellet after ultracentrifugation was resuspended in PBS (−) and was used as sEVs in subsequent experiments. Average particle size (z-average) of sEVs was determined with dynamic light scattering using a Zetasizer Nano ZS (Malvern Instruments, Malvern, U.K.) according to the manufacturer's protocol. One point five milliliter pooled healthy human plasma (10 healthy volunteers, range 18–53 years, median age 39.5 years; BioIVT, Westbury, NY, USA) was diluted eight-fold with PBS (−) and the sEVs-enriched fraction were prepared as described above [17].

### 2.7 Western blotting analysis

Cell protein was isolated from CRC cells by M-PER Mammalian Protein Extraction Reagent (Thermo Fisher Scientific, Waltham, MA, USA). Protein concentrations were was calculated by the Qubit™ Quantitation Fluorometer (Thermo Fisher Scientific). Following electrophoresis of 5 μg protein on 4–12% NuPAGE Bis-Tris gels (Invitrogen) with MES buffer, sample was transferred to PVDF membrane using iBlot (Invitrogen). PVDF membrane was incubated for 30 min with Blocking One (Nacalai Tesque) to block non-specific binding. The membrane was incubated with the primary antibody for 1 h at room temperature and was further incubated with the secondary antibody for 1 h at room temperature. The protein band was detected using Chemi-Lumi One Super (Nacalai Tesque). The antibodies were as follows: monoclonal mouse anti-heat shock 70 kDa protein (HSP70), monoclonal mouse anti-calnexin, goat anti-mouse IgG-HRP, and goat anti-rabbit IgG-HRP (Santa Cruz Biotechnology, Santa Cruz, CA, U.S.A.); polyclonal rabbit anti-CD9 and monoclonal mouse anti-Apolipoprotein A1 (APOA1) (Proteintech, Chicago, IL, U.S.A.).

### 2.8 Real-time RT-PCR for miRNA in sEVs

RNA in sEVs was extracted using Total Exosome RNA & Protein Isolation Kit (Invitrogen) according to the manufacturer's protocol. miScript Reverse Transcription Kit (QIAGEN, Hilden, Germany) was used for synthesis of cDNA from the maximum volume of RNA [23]. The amount of miRNAs was quantified by qPCR using the miScript SYBR Green PCR kit (QIAGEN). Normalization of miRNA amount was performed by miR-16–5p, using the 2^−ΔΔCt^ method [24]. The primer sequences were listed in Supplementary Table 1.

## 3 Results

### 3.1 Comparison of L-OHP sensitivity in CRC cells and establishment of L-OHP-resistant CRC cells

**Fig. 1:**
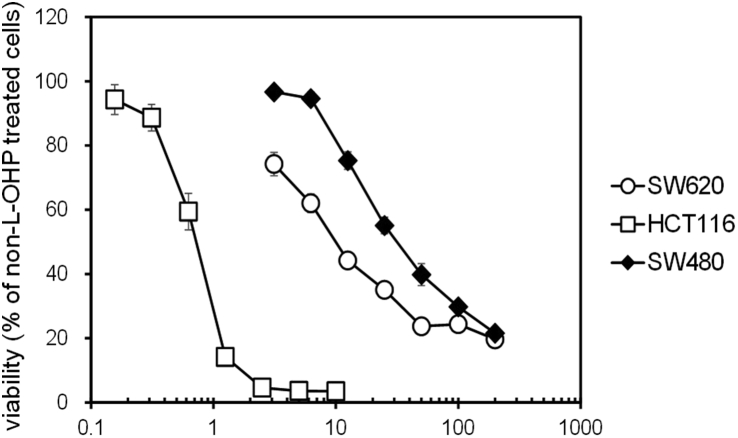
Comparison of the Sensitivity of CRC Cells to L-OHP. Cell viability of HCT116, SW620, and SW480 cells was measured by the WST-8 assay after L-OHP treatment for 72 h in a 96-well plate. L-OHP concentrations were 3.13, 6.25, 12.5, 25, 50, 100, 200 μM (SW620 and SW480 cells) or 0.16, 0.31, 0.63, 1.25, 2.5, 5, 10 μM (HCT116 cells). Each value represents the mean ± S.E.M. of three experiments.

First of all, we compared L-OHP sensitivity among CRC cell lines SW620, HCT116, and SW480. The IC_50_ value of L-OHP in SW480 cells was significantly and dramatically higher than SW620 and HCT116 cells (Table 1 and Fig. 1), suggesting the low sensitivity of SW480 cells to L-OHP. Therefore, in this study, SW480 cells were used as intrinsic L-OHP-resistant CRC cells. Secondly, in order to elucidate the effects of L-OHP resistance acquisition on miRNA expression, L-OHP-resistant CRC cells were established using sensitive CRC, SW620 and HCT116 cells, by long-term treatment with L-OHP. SW620-OxR cells were established in our previous report [9]. L-OHP-resistant HCT116 (HCT116-OxR) cells were newly established in this study. The IC_50_ value of L-OHP in HCT116 cells exposed to L-OHP for 145 days (41.44 ± 3.03 μM) was markedly higher than HCT116 cells (0.55 ± 0.05 μM). These cells were considered as acquired resistant cells for L-OHP.

**Table 1:** IC_50_ values of L-OHP in SW620, HCT116, and SW480 cells.

Cell line	IC_50_ (μM)
SW620	11.19 ± 1.37
HCT116	0.75 ± 0.03
SW480	35.53 ± 4.16**,^††^

The IC_50_ values of L-OHP in CRC cells were calculated as described in Materials and Methods. Each value represents the mean ± S.E.M. of three independent experiments (Student–Newman–Keuls test, **p < 0.01 significantly different from SW620 cells, ^††^*p* < 0.01significantly different from HCT116 cells).

### 3.2 Search for characteristic intracellular miRNA expression in L-OHP-resistant CRC cells

**Fig. 2:**
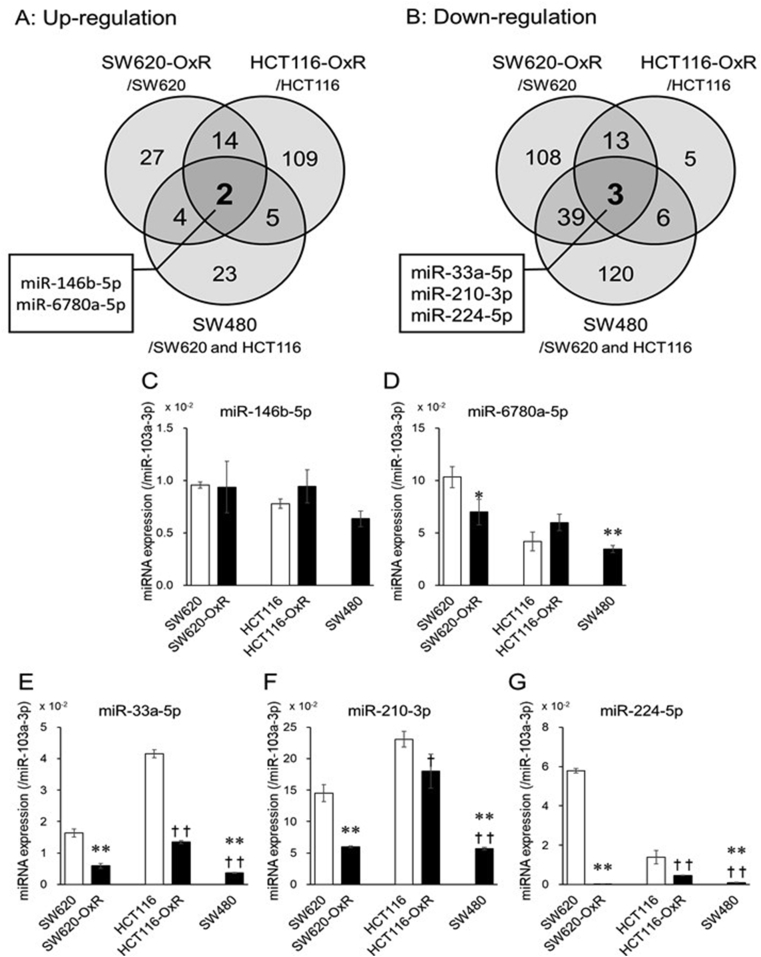
Intracellular miRNA expression levels in L-OHP-resistant CRC cells. Intracellular miRNA expression levels were normalized using miR-103a-3p. Venn diagram showed distribution of >1.5-fold high (A) or low (B) intracellular miRNA expressions in SW620 cells versus SW620/OxR cells, HCT116 cells versus HCT116/OxR cells, and SW620 and HCT116 cells versus SW480 cells. The expression levels of intracellular miR-146b-5p (C), miR-6780a-5p (D), miR-33a-5p (E), miR-210–3p (F), and miR-224–5p (G) were quantified by real-time RT-PCR. miR-103a-3p was used for normalization of intracellular miRNA expression levels. Each column represents the mean ± S.E.M. of three independent experiments (Student–Newman–Keuls test, *p < 0.05, **p < 0.01 significantly different from SW620 cells, ^†^*p* < 0.05, ^††^*p* < 0.01significantly different from HCT116 cells).

In order to find characteristic expression of intracellular miRNAs in L-OHP-resistant CRC cells, the intracellular miRNA expression of CRC cells was comprehensively analyzed by miRNA microarray analysis (supplementary material). As illustrated in Fig. 2A and B, the Venn diagram shows the number of miRNAs that were more than 1.5-fold differentially expressed in L-OHP-resistant cells compared with sensitive cells. The expression of miR-146b-5p and miR-6780a-5p was up-regulated and the expression of miR-33a-5p, miR-210–3p, and miR-224–5p was down-regulated in SW620-OxR, HCT116-OxR, and SW480 cells (Table 2, and Fig. 2A and B).

**Table 2:** Comparison of intracellular miRNA expression ratios in L-OHP-resistant CRC cells using microarray.

Cell	miRNA expression ratio (resistant cells/sensitive cells)				
	Up-regulation (>1.50)		Down-regulation (<0.67)		
	miR-146b-5p	miR-6780a-5p	miR-33a-5p	miR-210–3p	miR-224–5p
Acquired resistant cell					
SW620-OxR/SW620	8.63	7.40	0.35	0.35	<0.01
HCT116-OxR/HCT116	10.79	20.59	0.33	0.30	0.40
Intrinsic resistant cells					
SW480/SW620	9.46	15.64	0.66	0.40	<0.01
SW480/HCT116	8.38	13.85	0.65	0.13	0.01

Next, we tried to validate the intracellular miRNA expression level by real-time RT-PCR. Intracellular miR-146b-5p and miR-6780a-5p expression level was not up-regulated in L-OHP-resistant cells unlike the result of microarray (Fig. 2C and D). On the other hand, intracellular miR-33a-5p, miR-210–3p, and miR-224–5p were under-expressed in SW620-OxR and HCT116-OxR cells compared with each parental cell and these results were consistent with the those of microarray (Fig. 2E, F, and 2G). In addition, the intracellular expression level of those miRNAs was significantly lower in SW480 cells than SW620 and HCT116 cells (Fig. 2E, F, and 2G). Considering IC_50_ values of L-OHP in each type of CRC cells (Table 1), these results indicated that the lower expression of miR-33a-5p, miR-210–3p, and miR-224–5p could be related to the lower-sensitivity to L-OHP.

### 3.3 Effects of miRNAs expression on L-OHP sensitivity in CRC cells

**Fig. 3:**
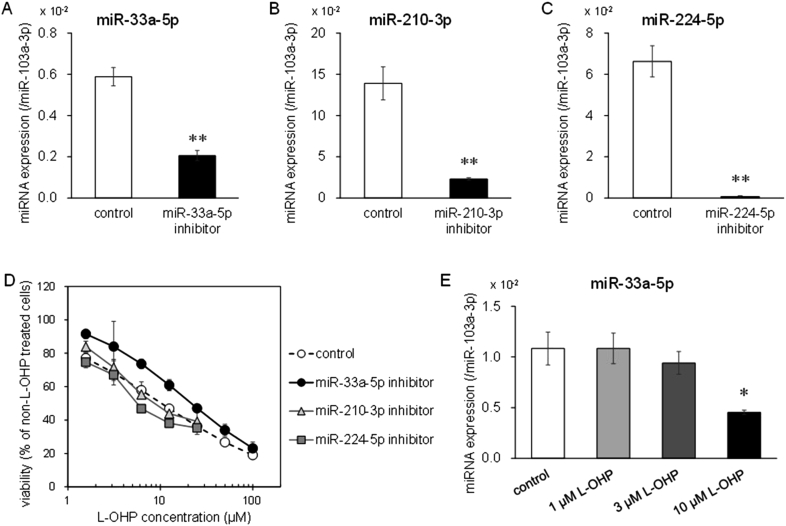
Effect of miRNA inhibitors on miRNAs expression in SW620 cells. After each inhibitor transfection for 72 h, intracellular miR-33a-5p (A), miR-210–3p (B), and miR-224–5p (C) expressions in SW620 cells transfected each miRNA inhibitor were detected using real time RT-PCR. The cells transfected with negative control inhibitor were used as control (Unpaired Student's t-test, **p < 0.01 significantly different from control). (D) After each inhibitor transfection for 72 h, SW620 cells were incubated with various concentrations of L-OHP (1.56, 3.13, 6.25, 12.5, 25, 50, 100 μM). Cell viability was measured by the WST-8 assay. (E) Intracellular miR-33a-5p expression was quantified in SW620 cells treated with L-OHP for 72 h. SW620 cells treated with dimethyl sulfoxide employed as control (Dunnett's test, *p < 0.05 significantly different from control). Each value represents the mean ± S.E.M. of three independent experiments.

The relationship between L-OHP sensitivity and expression of miR-33a-5p, miR-210–3p, or miR-224–5p was examined by transfection of miRNA inhibitors. Those intracellular miRNA expressions were decreased by those inhibitors in SW620 cells (Fig. 3A, B, and 3C). Furthermore, in SW620 cells, IC_50_ value of L-OHP was increased by miR-33a-5p inhibition (Table 3 and Fig. 3D). On the other hand, L-OHP sensitivity was not changed by miR-210–3p or miR-224–5p inhibitor in SW620 cells (Table 3 and Fig. 3D). Furthermore, intracellular miR-33a-5p expression was lower in surviving cells after treatment with 10 μM L-OHP than control cells (Fig. 3E). These results suggested that reduction of miR-33a-5p expression would result in acquisition of L-OHP resistance in CRC cells.

**Table 3:** IC_50_ values of L-OHP in SW620 cells that be transfected miRNA inhibitor.

	IC_50_ (μM)
control	9.90 ± 0.10
miR-33a-5p inhibitor	18.70 ± 3.13*
miR-210–3p inhibitor	10.72 ± 1.94
miR-224–5p inhibitor	7.15 ± 0.90

The IC_50_ value of L-OHP in SW620 cells transfected miRNA inhibitor was calculated as described in the Materials and Methods. Each value represents the mean ± S.E.M. of three independent experiments (Dunnett's test, *p < 0.05 significantly different from control cells).

### 3.4 Amount of extracellular miRNAs in sEVs secreted from L-OHP-resistant CRC cells

**Fig. 4:**
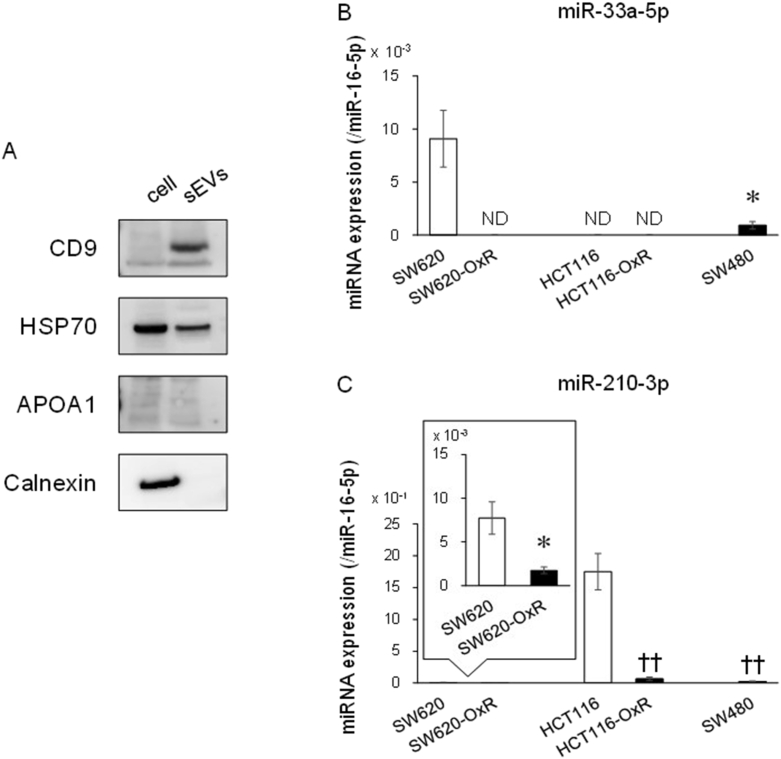
Extracellular miRNAs levels in sEVs secreted from L-OHP-resistant CRC cells. (A) The protein expression of sEVs secreted from SW620 cells was detected by Western blot analysis. CD9 and HSP70 were used as sEVs marker proteins. On the other hand, APOA1 and calnexin were used as non-sEVs marker proteins. (B, C) Amounts of miRNA in sEVs from CRC cells was detected using real-time RT-PCR and normalized by the amount of miR-16–5p. Each column represents the mean ± S.E.M. of three or four independent experiments (Student's *t*-test, *p < 0.05 significantly different from SW620 cells, Student–Newman–Keuls test, ^††^*p* < 0.01 significantly different from HCT116 cells). ND: Not detected.

From the above-mentioned results, extracellular miR-33a-5p, miR-210–3p, and miR-224–5p in sEVs secreted from cancer cells may be useful as non-invasive biomarker to predict L-OHP responses. Firstly, in order to confirm the successful isolation of sEVs by the method used in this study, we checked the characterization of sEVs such as the particle size and the presence of sEVs marker. The average particle size of sEVs isolated from cell culture medium was 78.4 ± 1.2 nm. The presence of CD9 and HSP70, sEVs marker proteins, in sEVs, was confirmed (Fig. 4A). In addition, APOA1 and calnexin, a non-sEVs marker protein, was not detected in sEVs (Fig. 4A). Thus, sEVs were found to be successfully isolated from culture medium.

Next, the amount of miR-33a-5p, miR-210–3p, and miR-224–5p in isolated sEVs was examined using real-time RT-PCR. It was first indicated that the amount of miR-33a-5p and miR-210–3p in sEVs secreted from L-OHP-resistant CRC cells tended to be smaller than sensitive CRC cells (Fig. 4B and C), consistent with the results of intracellular miRNA levels (Fig. 2E and F). However, miR-33a-5p was not detected in sEVs secreted from HCT116 and HCT116-OxR cells (Fig. 4B). In addition, the amount of miR-210–3p in sEVs secreted from SW620 and SW620-OxR cells was very small compared with other cells (Fig. 4C). miR-224–5p was not detected in sEVs secreted by CRC cells examined (data not shown).

## 4 Discussion

Results obtained in the present study first suggested that miR-33a-5p in sEVs may be potentially useful as non-invasive biomarker for L-OHP sensitivity. In addition, the amount of miR-210–3p in sEVs secreted from L-OHP-resistant cells was small. Furthermore, considering that miR-33a-5p and miR-210–3p were not detected in sEVs-enriched fraction isolated from healthy human serum (data not shown), the detection of miR-33a-5p and/or miR-210–3p in serum suggests that CRC cells would be present in the body. In addition, the decrease of these miRNAs amount in serum of CRC patients probably indicate L-OHP resistance acquisition and can be non-invasively monitored during the treatment with L-OHP.

As shown in Table 3, intracellular miR-33a-5p would make cancer cells more sensitive to L-OHP, but the details remain unknown. The inhibition of several genes by miR-33a-5p in L-OHP-resistant CRC cells would be one of possible explanations. For example, *PIM1* was reported to be suppressed by miR-33a-5p [25,26]. Besides, the overexpression of *PIM1* resulted in resistance to L-OHP-induced DNA damage [27]. Similarly, *HIF1A* was miR-33a-5p's target and inhibited L-OHP effects [28,29]. Therefore, it is quite possible that these genes were inversely increased by the suppression of miR-33a-5p, leading to the acquisition of resistance.

Extracellular miR-210–3p amount in sEVs secreted from two acquired L-OHP-resistant cells was smaller than that from each parental cell (Fig. 2C), suggesting candidate of the biomarker suitable for monitoring of L-OHP sensitivity during chemotherapy. The amount of miR-33a-5p or miR-210–3p was very small in sEVs from HCT116 or SW620 cells, respectively (Fig. 2B and C), possibly due to the abnormal mechanisms of sorting specific miRNAs into sEVs [[30], [31], [32]]. These results suggested that it is insufficient to determine L-OHP sensitivity by miR-33a-5p or miR-210–3p alone. However, recent studies had shown that the combination of several biomarkers would be a useful strategy to overcome this limitation [[33], [34], [35]]. Therefore, further studies for searching additional biomarkers and the optimal combination of several biomarkers, including miR-33a-5p and miR-210–3p, are needed. Furthermore, cohort analysis was required for demonstrating the importance of these miRNAs in CRC patients.

In conclusion, this study is the first report that miR-33a-5p and/or miR-210–3p in acquired and intrinsic L-OHP-resistant CRC cells and sEVs secreted from these cells were under-expressed. In particular, miR-33a-5p is a probable candidate for non-invasive biomarker of L-OHP sensitivity and also would enhance L-OHP sensitivity of CRC cells. These observations may serve to non-invasively identify acquired and intrinsic L-OHP-resistant CRC patients in the future.

## Declaration of competing interest

The authors declare that they have no known competing financial interests or personal relationships that could have appeared to influence the work reported in this paper.
