# Supplementary material for: miR-33a-5p in small extracellular vesicles as non-invasive biomarker for oxaliplatin sensitivity in human colorectal cancer cells
Source: Biochem Biophys Rep. 2021 Apr 7;26:100996. doi: 10.1016/j.bbrep.2021.100996 (PMC8058522; doi:10.1016/j.bbrep.2021.100996)
Supplement: Multimedia component 2 [file mmc2.docx]

Supplementary Table 1. Real-time RT-PCR Primers for miRNA Expression

| miRNA | Primer (5’-3’) |
| --- | --- |
| miR-16-5p | TAGCAGCACGTAAATATTGGCG |
| miR-33a-5p | GTGCATTGTAGTTGCATTGCA |
| miR-103a-3p | AGCAGCATTGTACAGGGCTATGA |
| miR-146b-5p | TGAGAACTGAATTCCATAGGCTG |
| miR-210-3p | CTGTGCGTGTGACAGCGGCTGA |
| miR-224-5p | TCAAGTCACTAGTGGTTCCGTTTAG |
| miR-6780b-5p | TTGGGAGGGAAGACAGCTGGAGA |

Supplemental figure legends

Supplemental Fig. S1. Microarrayanalysis of intracellular miRNA expressions.

Intracellular miRNA expression levels were normalized using miR-103a-3p. Venn diagram showed distribution of >1.5-fold high (A) or low (B) intracellular miRNA expressions in SW620 cells versus SW620/OxR cells, HCT116 cells versus HCT116/OxR cells, and SW620 and HCT116 cells versus SW480 cells.

Supplemental Fig. S2. Effect of miRNA inhibitors on miRNAs expression in SW620 cells.

After miR-33a-5p, miR-210-3p, and miR-224-5p inhibitor transfection for 72-h, each intracellular miRNA expression in SW620 cells was detected using real time RT-PCR. Each column represents the mean ± S.E.M. of three independent experiments (Unpaired Student’s *t*-test, ***p* <0.01 significantly different from SW620 cells transfected with control).

Supplemental Fig. S1

Supplemental Fig. S2
